# Supplementary material for: Potential role of developmental experience in the emergence of the parvo-magno distinction
Source: Commun Biol. 2025 Jul 3;8:987. doi: 10.1038/s42003-025-08382-4 (PMC12229566; doi:10.1038/s42003-025-08382-4)
Supplement: Supplementary file 1 — Supplemental Material PDF [file 42003_2025_8382_MOESM1_ESM.pdf]

Supplementary Material for:
Potential role of developmental experience in the emergence of the parvo-magno distinction

Marin Vogelsang, Lukas Vogelsang, Gordon Pipa, Sidney Diamond, Pawan Sinha

This PDF includes:

Supplementary Figs. 1-22

- Supplementary Fig. 1: Overview of training settings and regimens
- Supplementary Figs. 2-12: Additional RF plots for setting 1 (2), setting 2 (3-4), setting 3 (5-6), setting 4 (7-8), setting 5 (9-10), and biomimetic regimens v2-4 within setting 1 (11-12)
- Supplementary Figs. 13-17: Shape/texture plots for setting 2 (13), setting 3 (14), setting 4 (15), setting 5 (16), biomimetic regimens v2-4 within setting 1 (17), and setting 1 (18), including with ablation of high vs. low spatial frequency units (rather than only ablation of high vs. low color units).
- Supplementary Fig. 19: Visualization of the least temporally-varied RFs (3D CNNs)
- Supplementary Fig. 20: RF plots for all 5 random initializations (3D CNNs)
- Supplementary Fig. 21: Training/validation loss and accuracy for all trained models
- Supplementary Fig. 22: Comparison of our color metric with computations in other color spaces

Supplementary Table 1

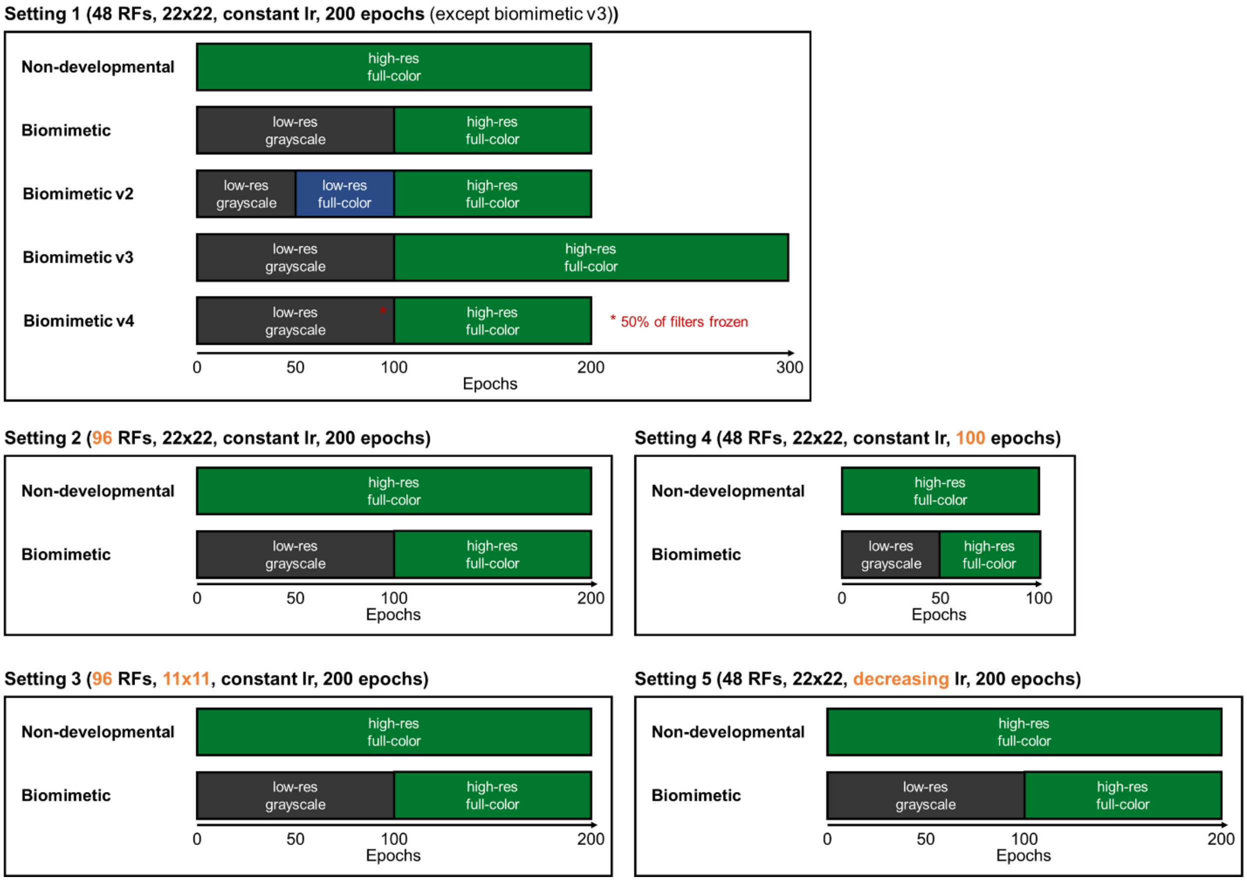

Supplementary Fig. 1. Illustration of the different settings and regimens used for training our 2D networks.

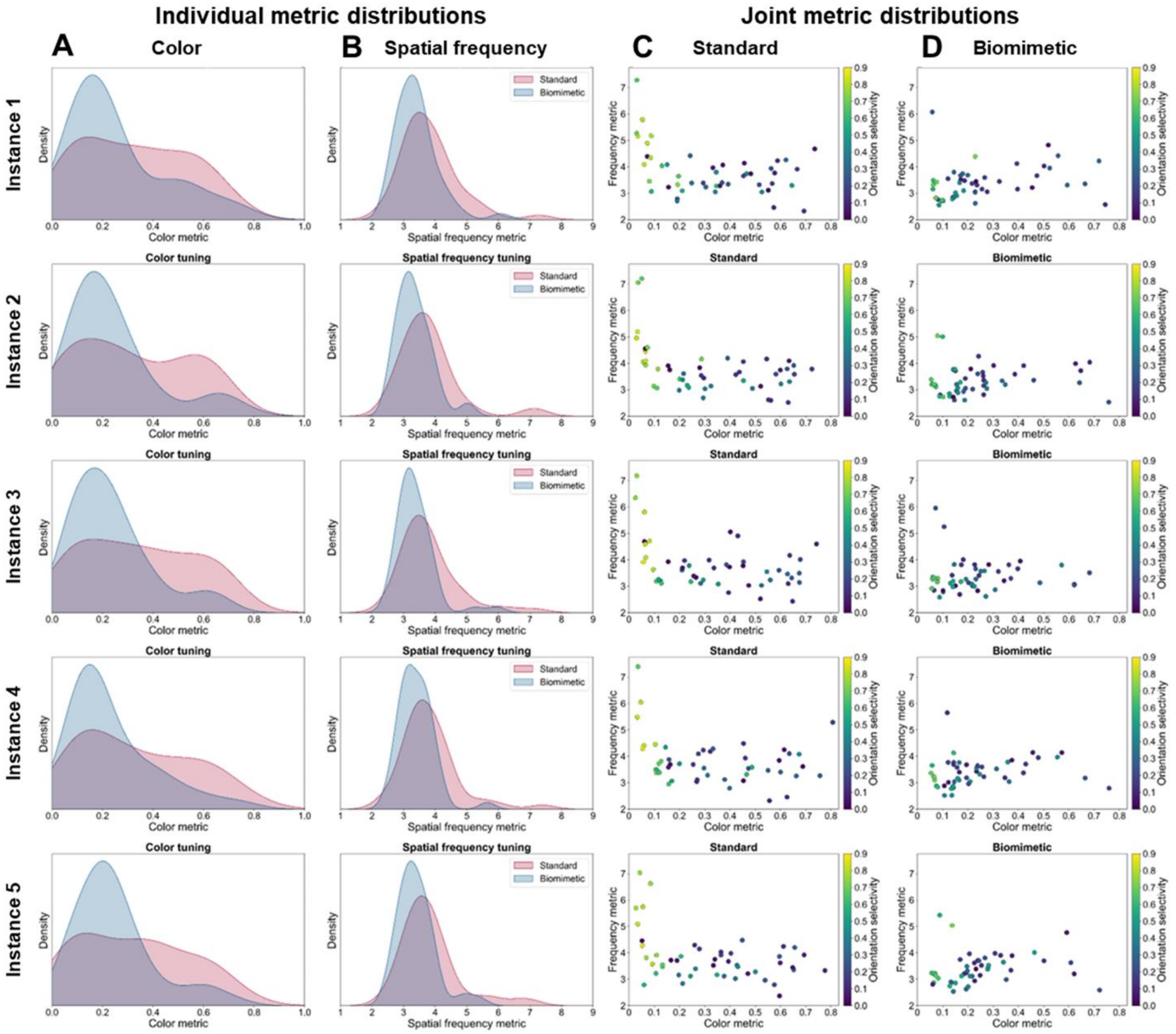

Supplementary Fig. 2. Depiction of individual and joint metric distributions for setting 1 (48 22x22 pixel RFs) across all five training runs with different random initializations (the first run is shown in Figure 1 in the main manuscript). **A&B.** Color and spatial frequency distributions of individual RFs. **C&D.** Scatter plots depicting the joint frequency and color coding of individual RFs.

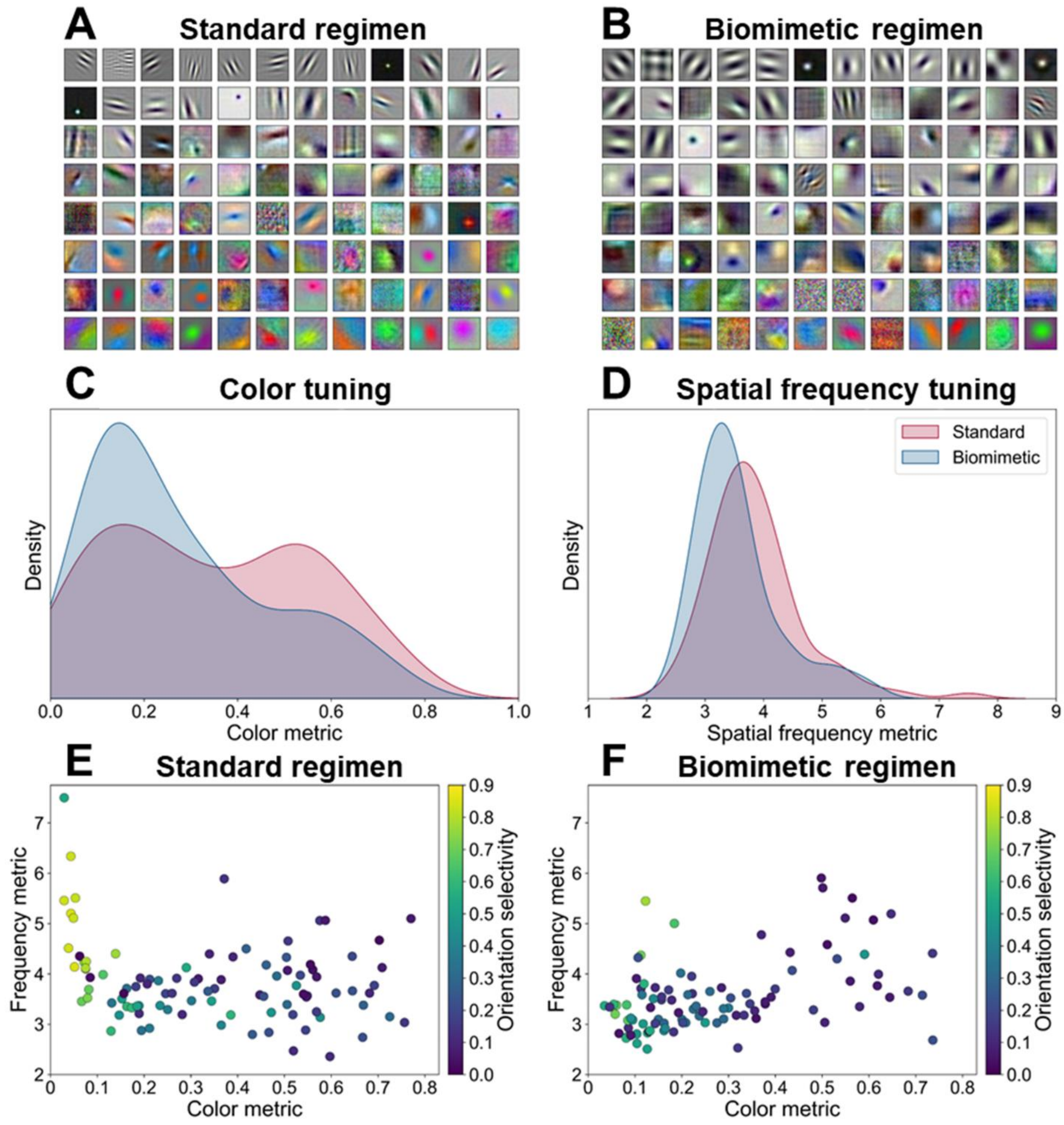

Supplementary Fig. 3. Reproduction of Figure 1 in the main manuscript when utilizing setting 2 (96 22x22 pixel RFs). **A&B.** Visualization of first-layer RFs. **C&D.** Color and spatial frequency distributions of individual RFs. **E&F.** Scatter plots depicting the joint frequency and color coding of individual RFs. Depicted here are results obtained with the first training run; outcomes of five training runs with different random initializations are shown in Supplementary Figure 4.

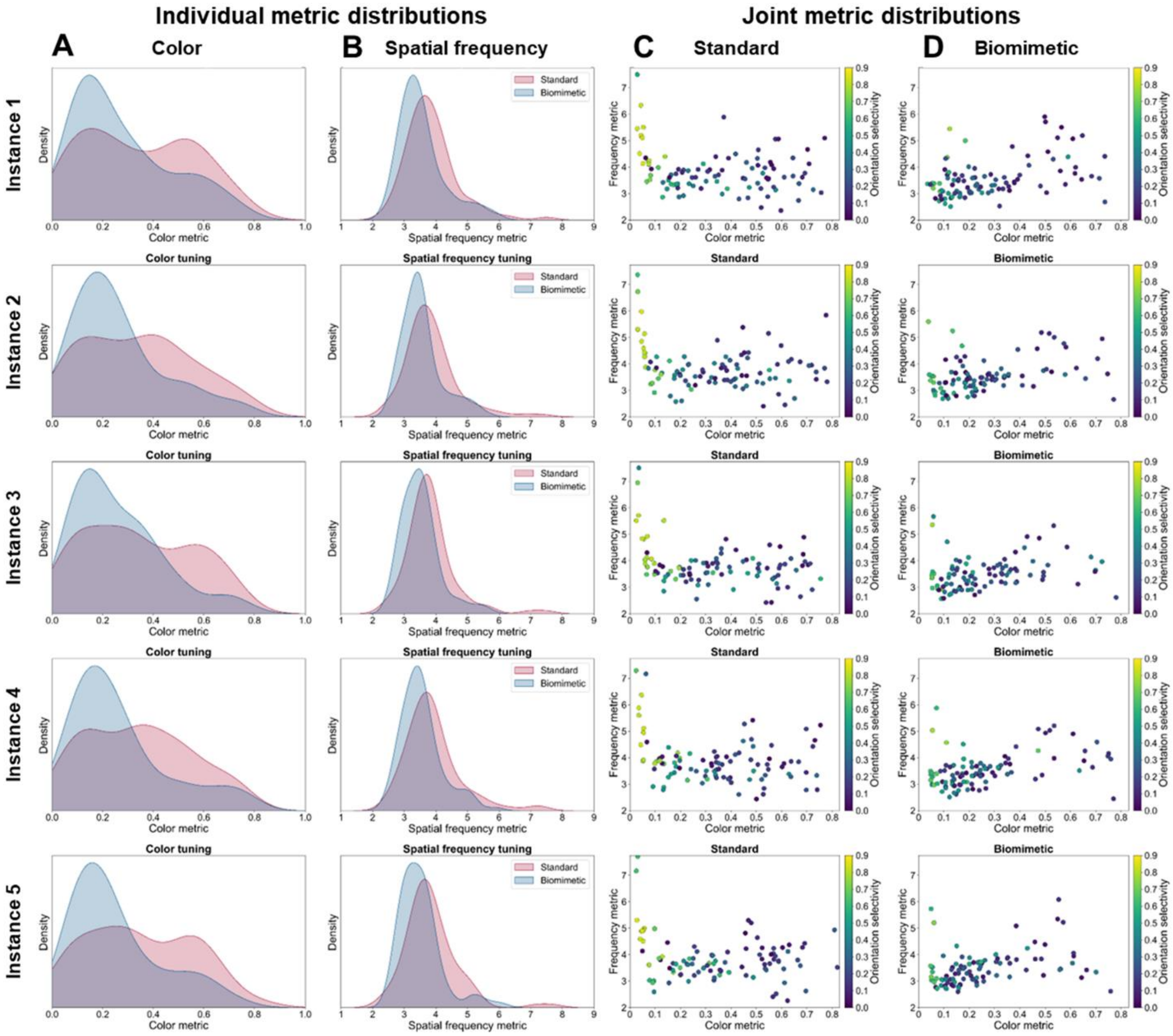

Supplementary Fig. 4. Depiction of individual and joint metric distributions for setting 2 (96 22x22 pixel RFs) across all five training runs with different random initializations (the first run is shown in Supplementary Figure 3). **A&B.** Color and spatial frequency distributions of individual RFs. **C&D.** Scatter plots depicting the joint frequency and color coding of individual RFs.

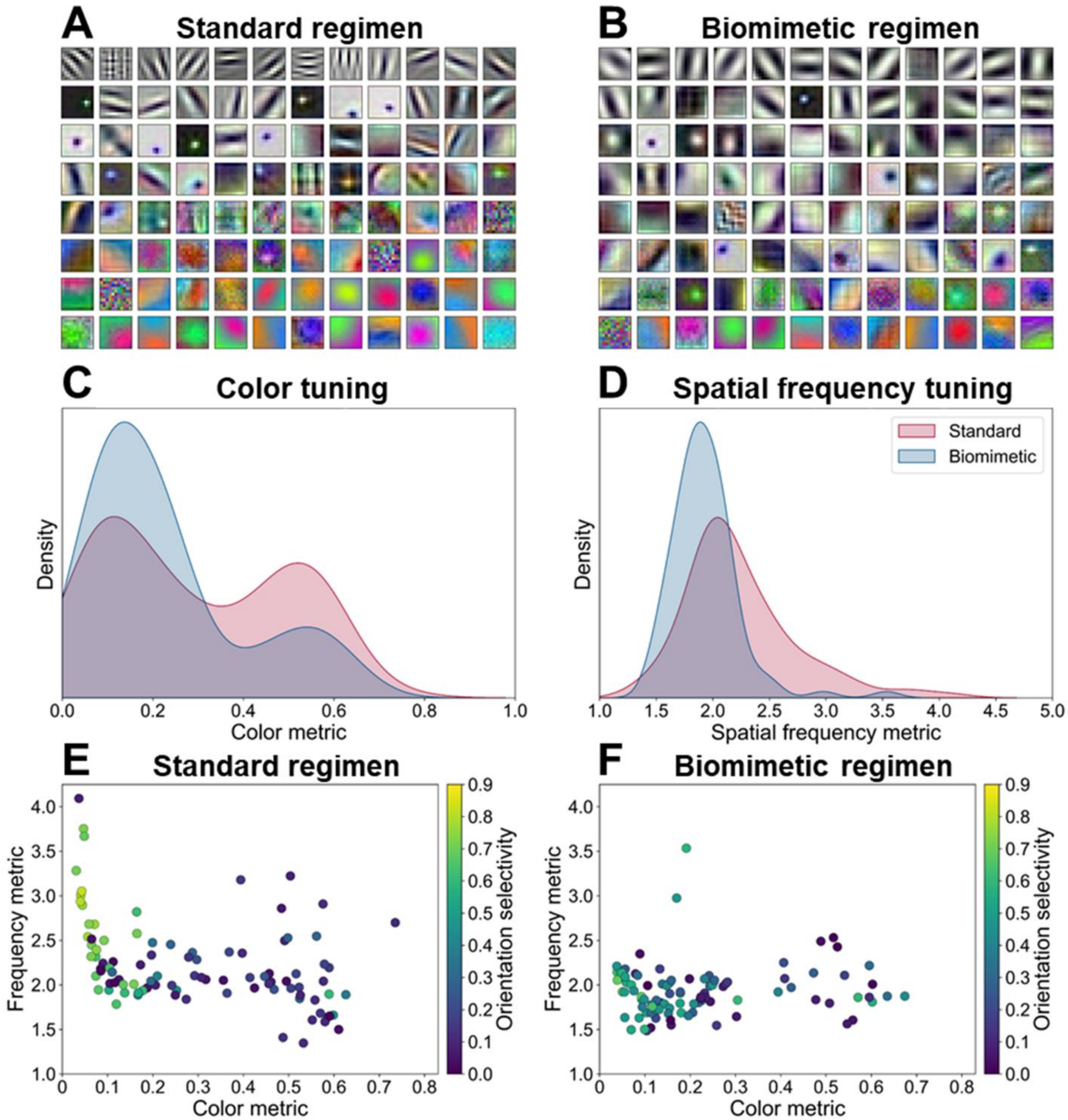

Supplementary Fig. 5. Reproduction of Figure 1 in the main manuscript when utilizing setting 3 (96 11x11 pixel RFs). **A&B.** Visualization of first-layer RFs. **C&D.** Color and spatial frequency distributions of individual RFs. **E&F.** Scatter plots depicting the joint frequency and color coding of individual RFs. Depicted here are results obtained with the first training run; outcomes of five training runs with different random initializations are shown in Supplementary Figure 6.

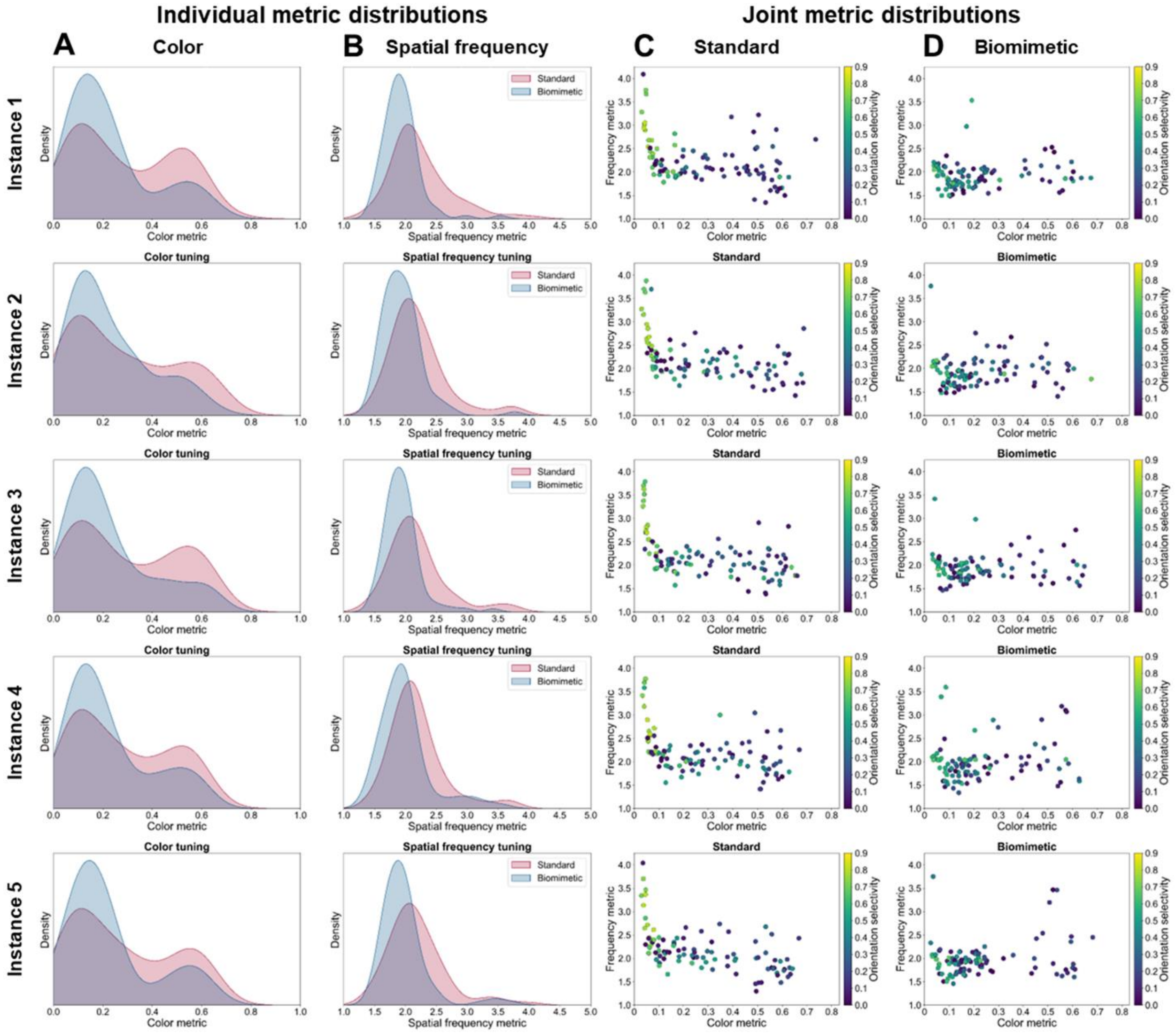

Supplementary Fig. 6. Depiction of individual and joint metric distributions for setting 3 (96 11x11 pixel RFs) across all five training runs with different random initializations (the first run is shown in Supplementary Figure 5). **A&B.** Color and spatial frequency distributions of individual RFs. **C&D.** Scatter plots depicting the joint frequency and color coding of individual RFs.

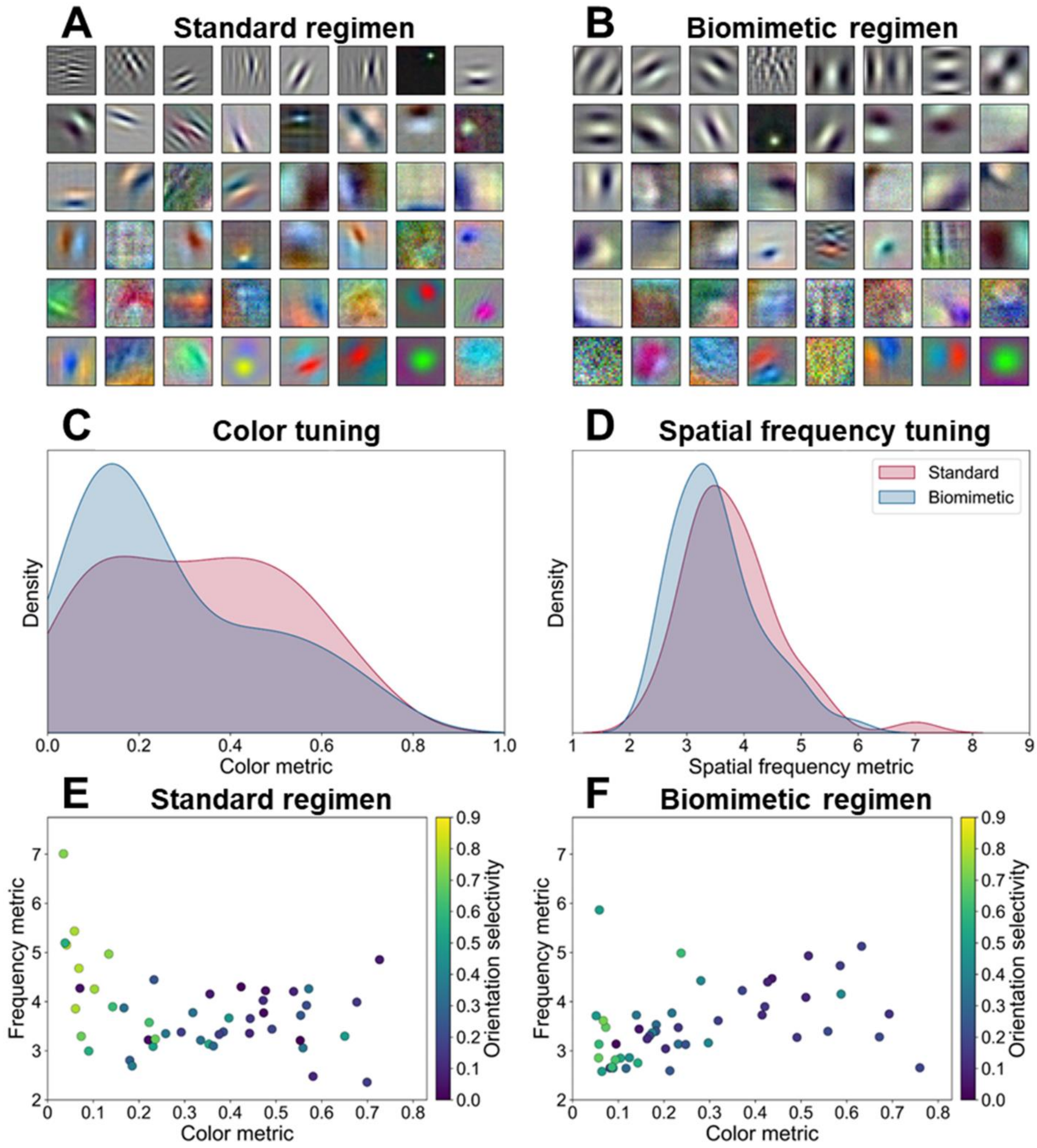

Supplementary Fig. 7. Reproduction of Figure 1 in the main manuscript when utilizing setting 4 (fewer epochs). **A&B.** Visualization of first-layer RFs. **C&D.** Color and spatial frequency distributions of individual RFs. **E&F.** Scatter plots depicting the joint frequency and color coding of individual RFs. Depicted here are results obtained with the first training run; outcomes of five training runs with different random initializations are shown in Supplementary Figure 8.

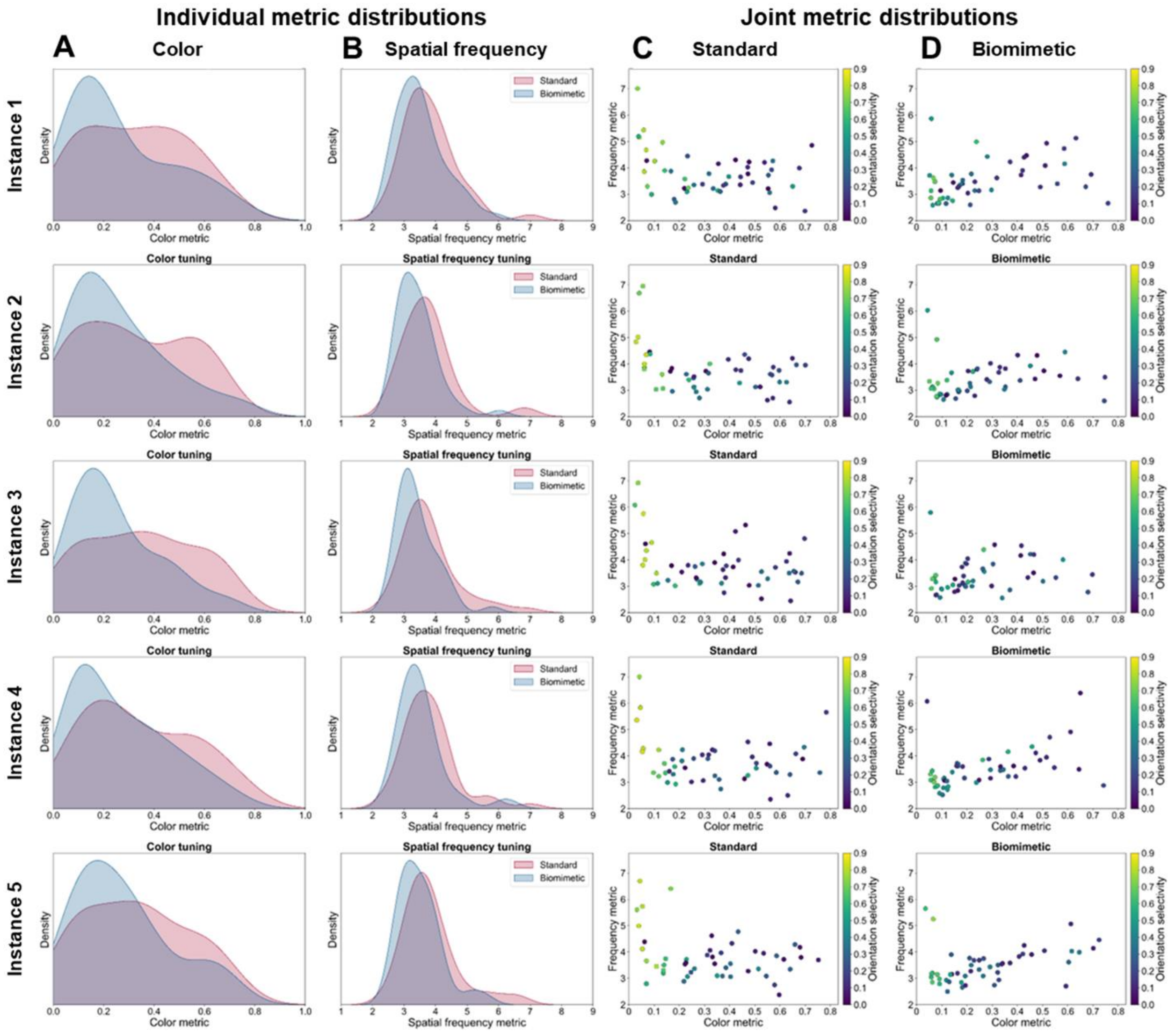

Supplementary Fig. 8. Depiction of individual and joint metric distributions for setting 4 (fewer epochs) across all five training runs with different random initializations (the first run is shown in Supplementary Figure 7). **A&B.** Color and spatial frequency distributions of individual RFs. **C&D.** Scatter plots depicting the joint frequency and color coding of individual RFs.

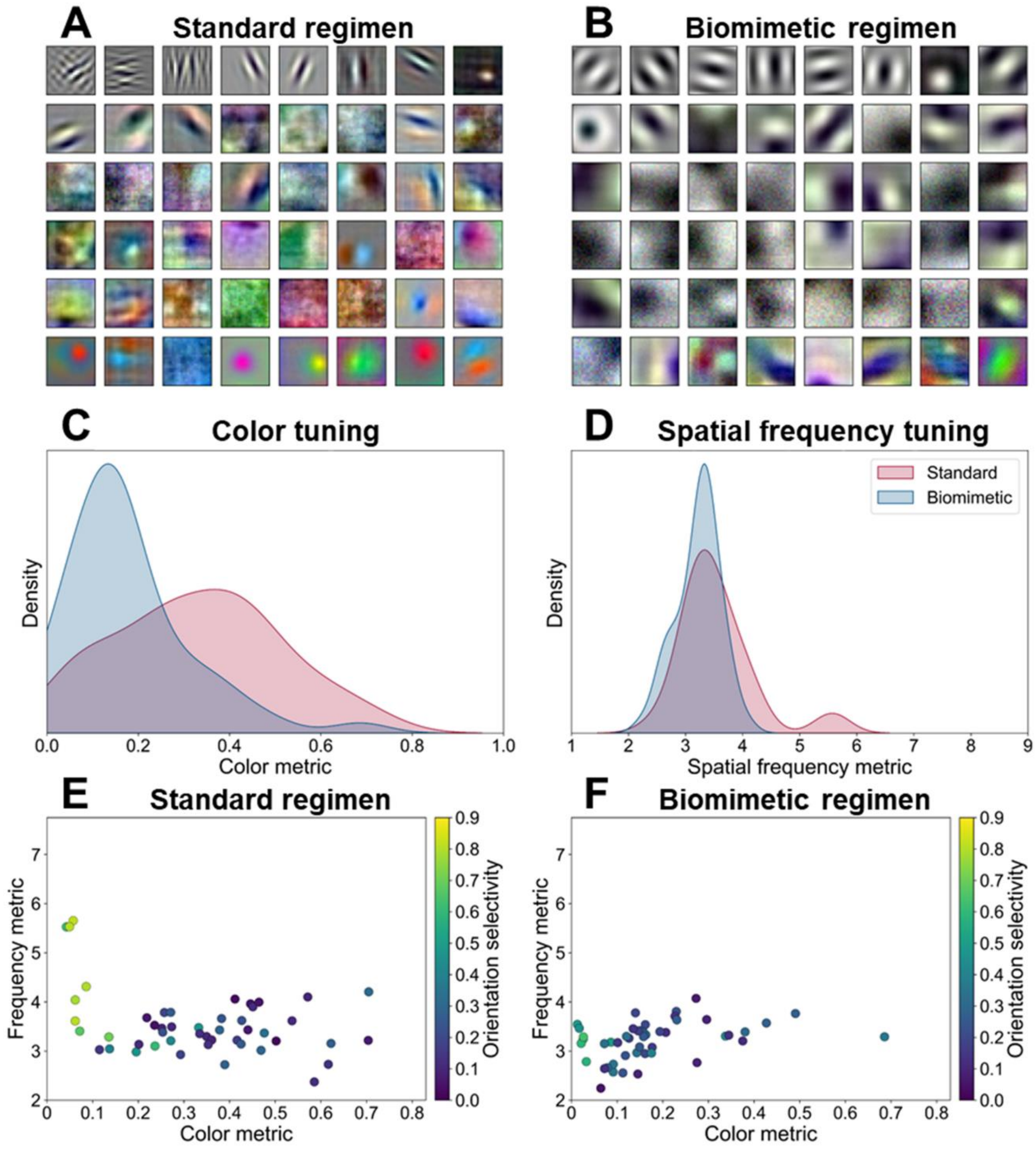

Supplementary Fig. 9. Reproduction of Figure 1 in the main manuscript when utilizing setting 5 (decreasing learning rate). **A&B.** Visualization of first-layer RFs. **C&D.** Color and spatial frequency distributions of individual RFs. **E&F.** Scatter plots depicting the joint frequency and color coding of individual RFs. Depicted here are results obtained with the first training run; outcomes of five training runs with different random initializations are shown in Supplementary Figure 10.

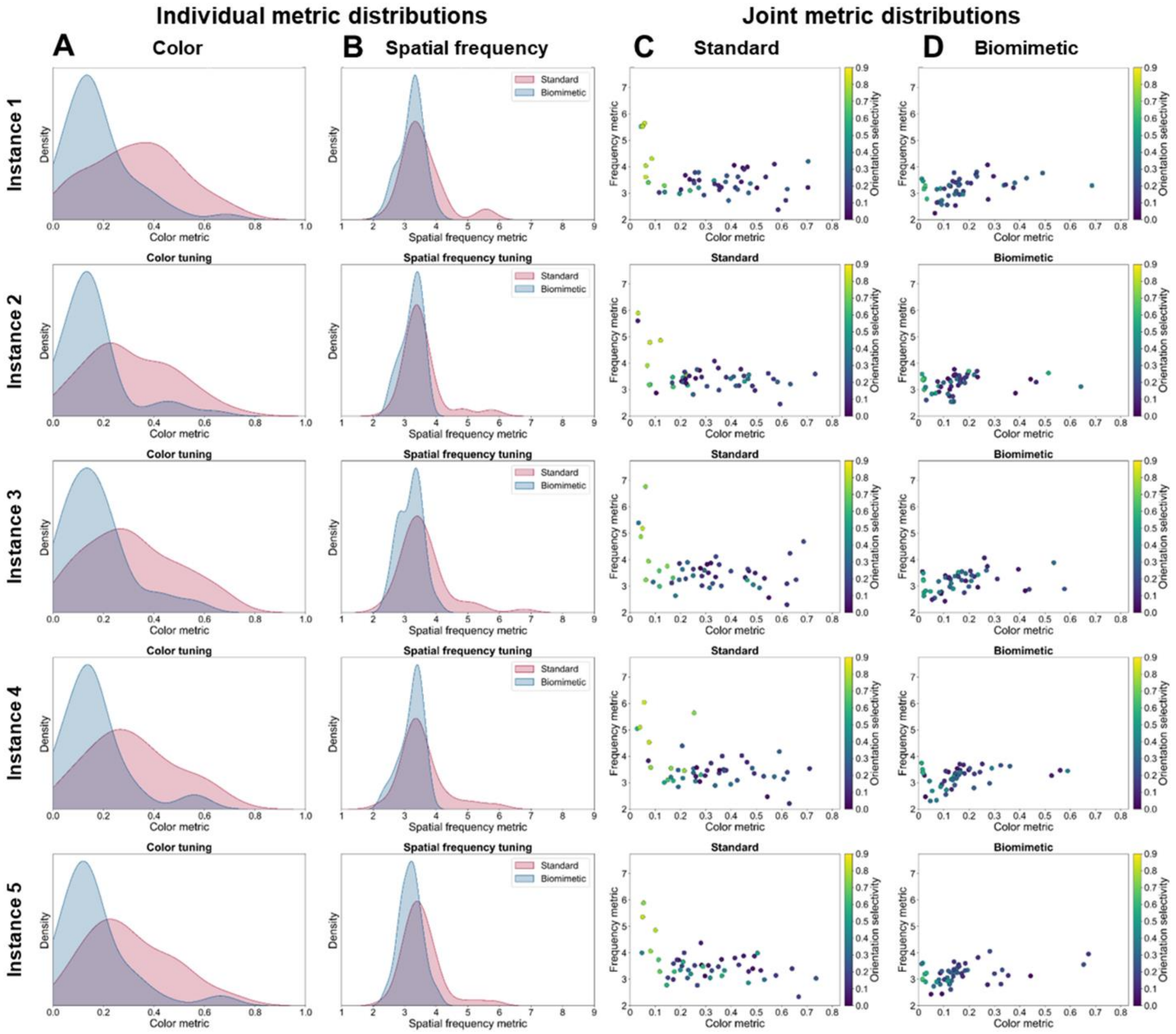

Supplementary Fig. 10. Depiction of individual and joint metric distributions for setting 5 (decreasing learning rate) across all five training runs with different random initializations (the first run is shown in Supplementary Figure 9). **A&B.** Color and spatial frequency distributions of individual RFs. **C&D.** Scatter plots depicting the joint frequency and color coding of individual RFs.

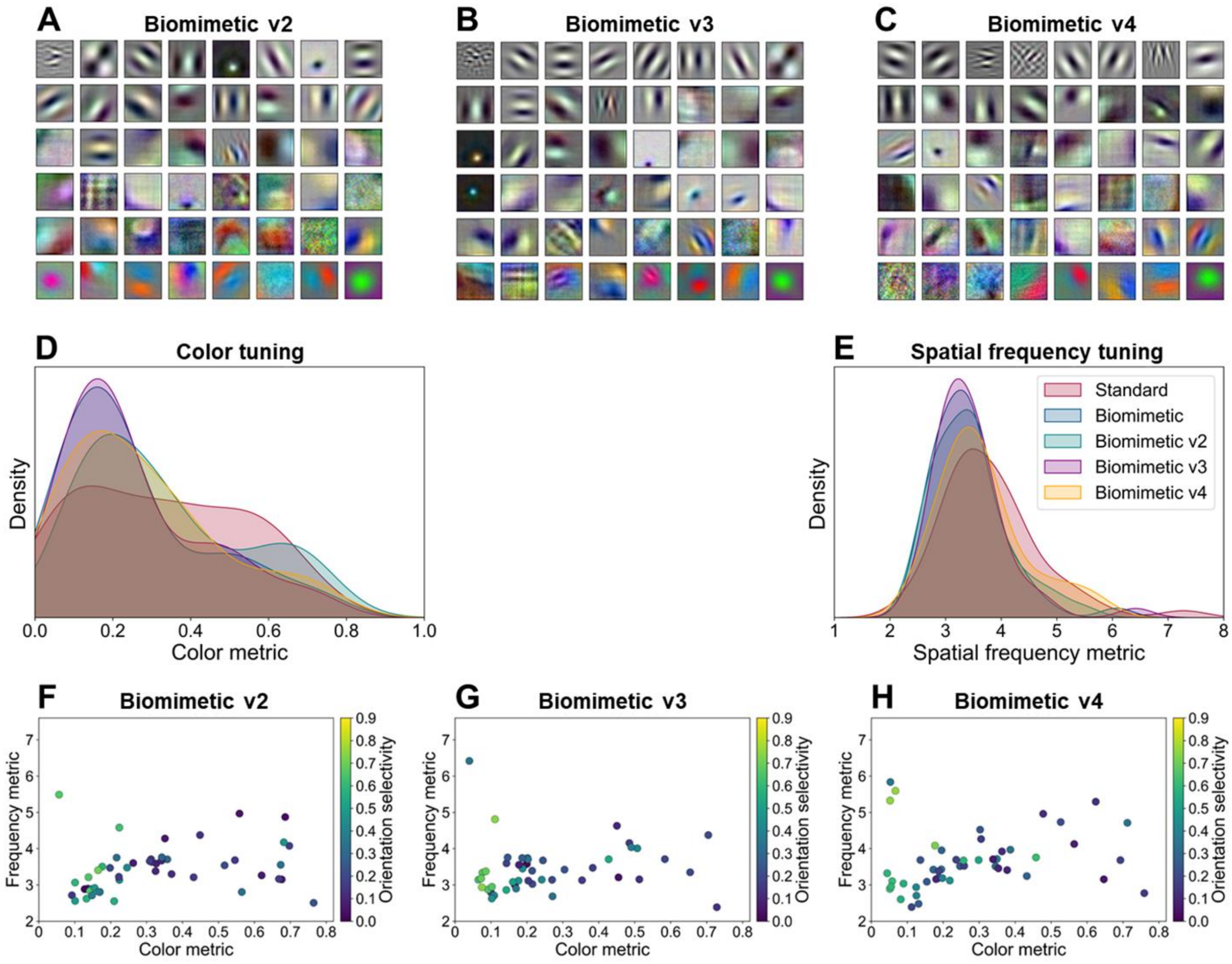

Supplementary Fig. 11. Reproduction of Figure 1 in the main manuscript for biomimetic regimens v2-v4 within setting 1 (48 22x22 pixel RFs). **A-C.** Visualization of first-layer RFs. **D&E.** Color and spatial frequency distributions of individual RFs. **F-H.** Scatter plots depicting the joint frequency and color coding of individual RFs. Depicted here are results obtained with the first training run; outcomes of five training runs with different random initializations are shown in Supplementary Figure 12.

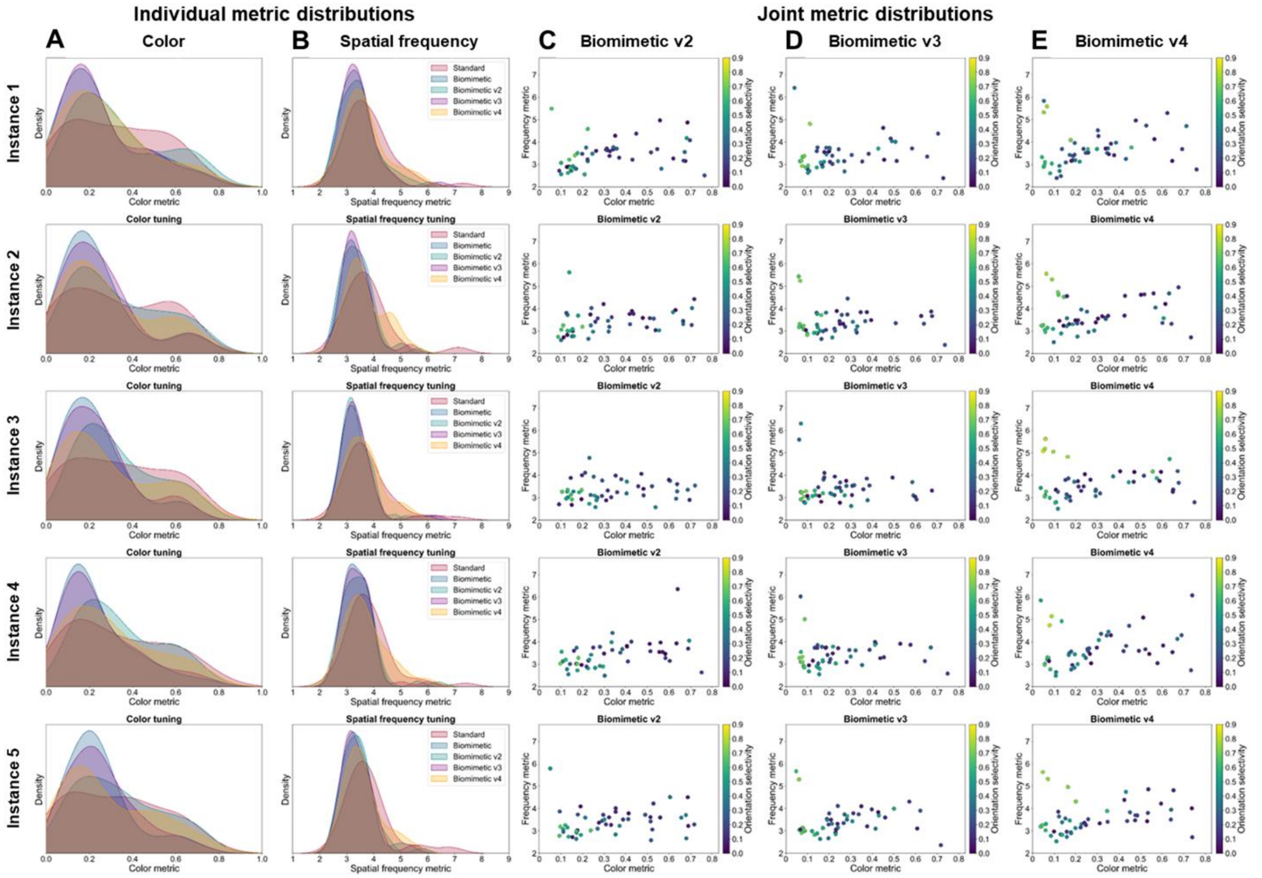

Supplementary Fig. 12. Depiction of individual and joint metric distributions for biomimetic v2-v4 regimens within setting 1 (48 22x22 pixel RFs) across all five training runs with different random initializations (the first run is shown in Supplementary Figure 11). **A&B.** Color and spatial frequency distributions of individual RFs. **C-E.** Scatter plots depicting the joint frequency and color coding of individual RFs.

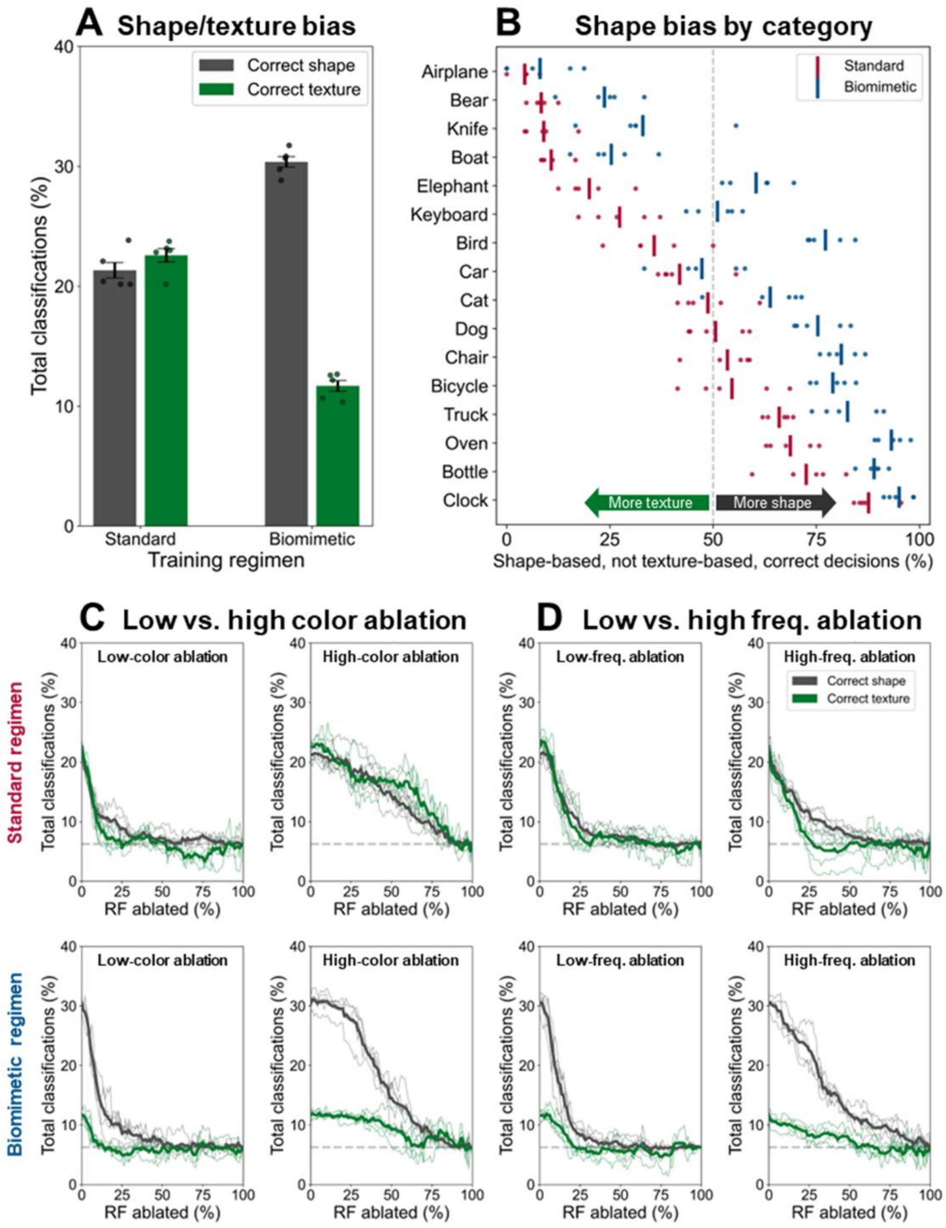

Supplementary Fig. 13. Reproduction and extension of Figure 2 in the main manuscript when utilizing setting 2 (96 22x22 pixel RFs). **A.** Percentage of total classifications correct in terms of shape, correct in terms of texture, or incorrect. Error bars represent the standard error across the five different training runs, and dots depict results of each individual run. **B.** Percentage of shape-based correct classifications, as opposed to texture-based correct classifications, for each of the 16 different super-classes used. Shown here are results of the five individual training runs (dots), along with their means (bars). **C&D.** Shape/texture bias as a function of the proportion of ablated units with lowest color, highest color, lowest frequency, and highest frequency (from left to right) tuning. Depicted are the five training runs (thin lines) as well as their means (thick lines).

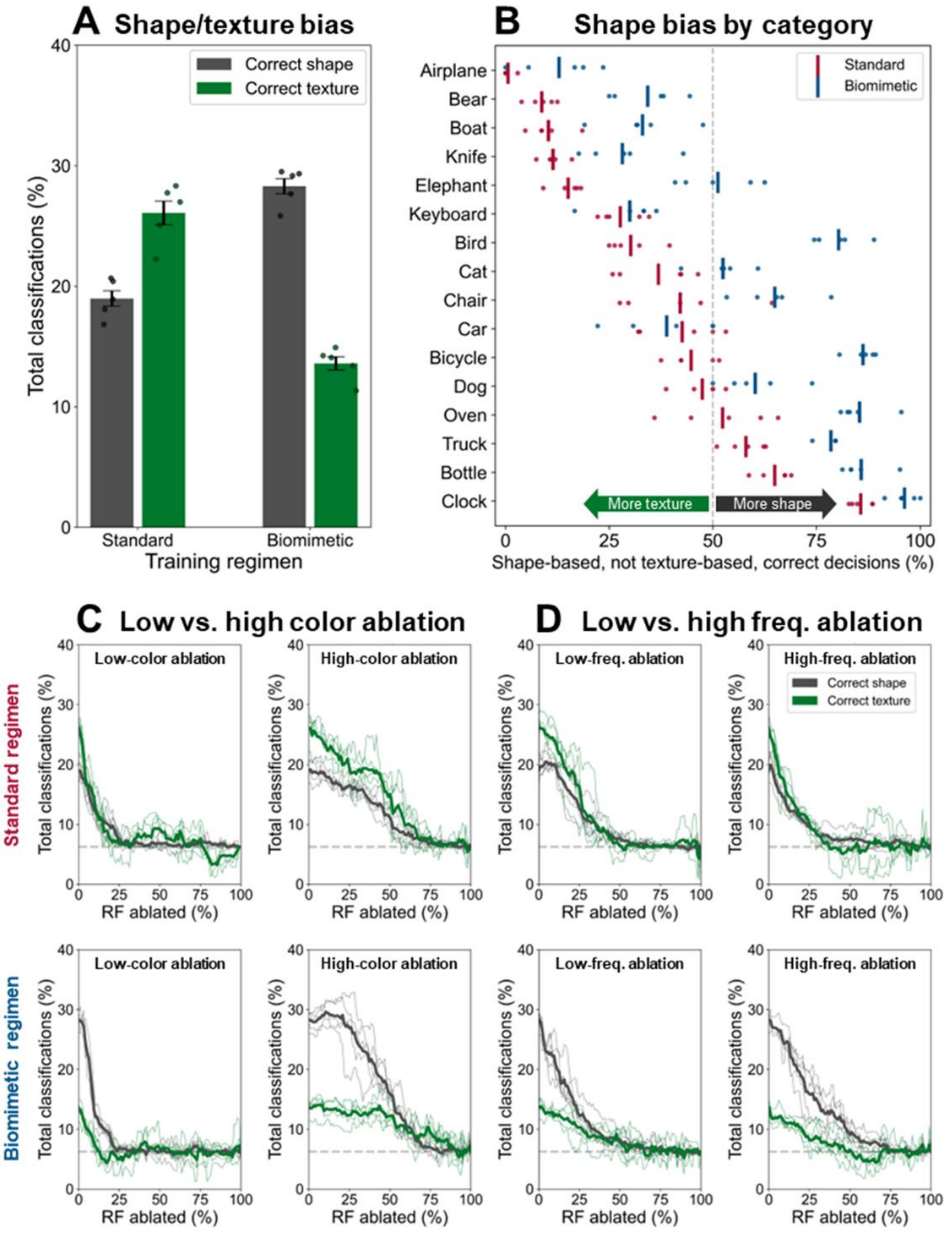

Supplementary Fig. 14. Reproduction and extension of Figure 2 in the main manuscript when utilizing setting 3 (96 11x11 pixel RFs). **A.** Percentage of total classifications correct in terms of shape, correct in terms of texture, or incorrect. Error bars represent the standard error across the five different training runs, and dots depict results of each individual run. **B.** Percentage of shape-based correct classifications, as opposed to texture-based correct classifications, for each of the 16 different super-classes used. Shown here are results of the five individual training runs (dots), along with their means (bars). **C&D.** Shape/texture bias as a function of the proportion of ablated units with lowest color, highest color, lowest frequency, and highest frequency (from left to right) tuning. Depicted are the five training runs (thin lines) as well as their means (thick lines).

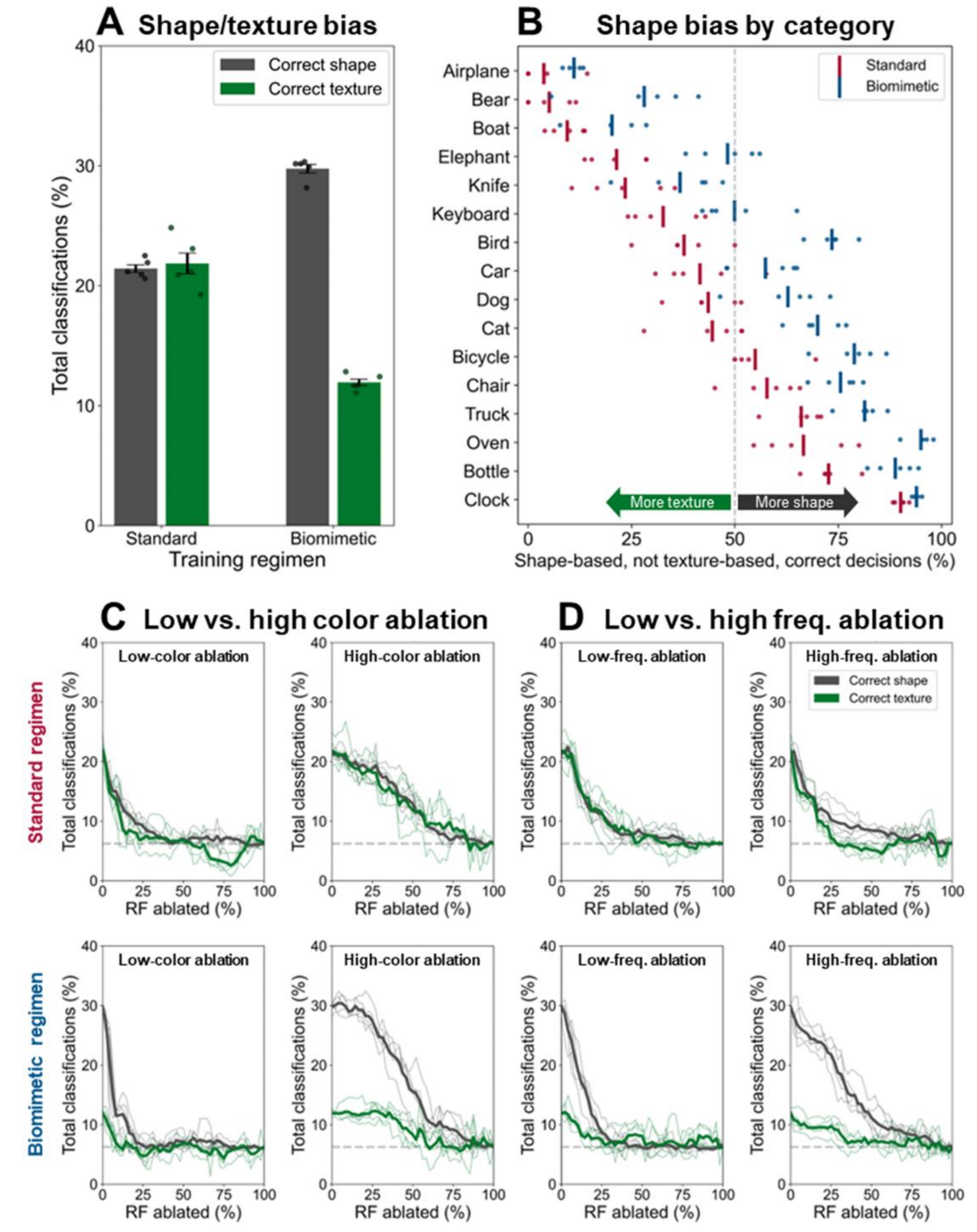

Supplementary Fig. 15. Reproduction and extension of Figure 2 in the main manuscript when utilizing setting 4 (fewer epochs). **A.** Percentage of total classifications correct in terms of shape, correct in terms of texture, or incorrect. Error bars represent the standard error across the five different training runs, and dots depict results of each individual run. **B.** Percentage of shape-based correct classifications, as opposed to texture-based correct classifications, for each of the 16 different super-classes used. Shown here are results of the five individual training runs (dots), along with their means (bars). **C&D.** Shape/texture bias as a function of the proportion of ablated units with lowest color, highest color, lowest frequency, and highest frequency (from left to right) tuning. Depicted are the five training runs (thin lines) as well as their means (thick lines).

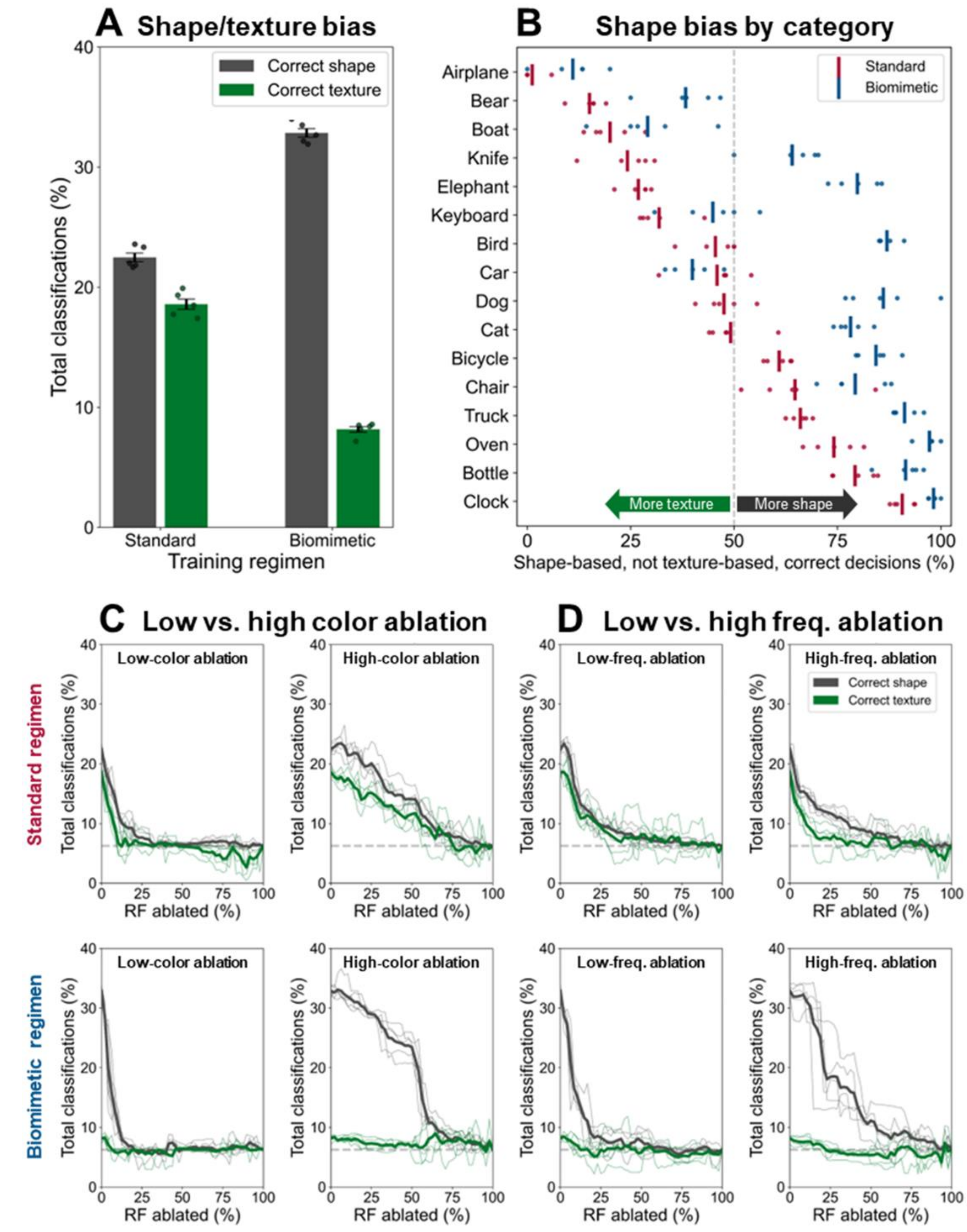

Supplementary Fig. 16. Reproduction and extension of Figure 2 in the main manuscript when utilizing setting 5 (decreasing learning rate). **A.** Percentage of total classifications correct in terms of shape, correct in terms of texture, or incorrect. Error bars represent the standard error across the five different training runs, and dots depict results of each individual run. **B.** Percentage of shape-based correct classifications, as opposed to texture-based correct classifications, for each of the 16 different super-classes used. Shown here are results of the five individual training runs (dots), along with their means (bars). **C&D.** Shape/texture bias as a function of the proportion of ablated units with lowest color, highest color, lowest frequency, and highest frequency (from left to right) tuning. Depicted are the five training runs (thin lines) as well as their means (thick lines).

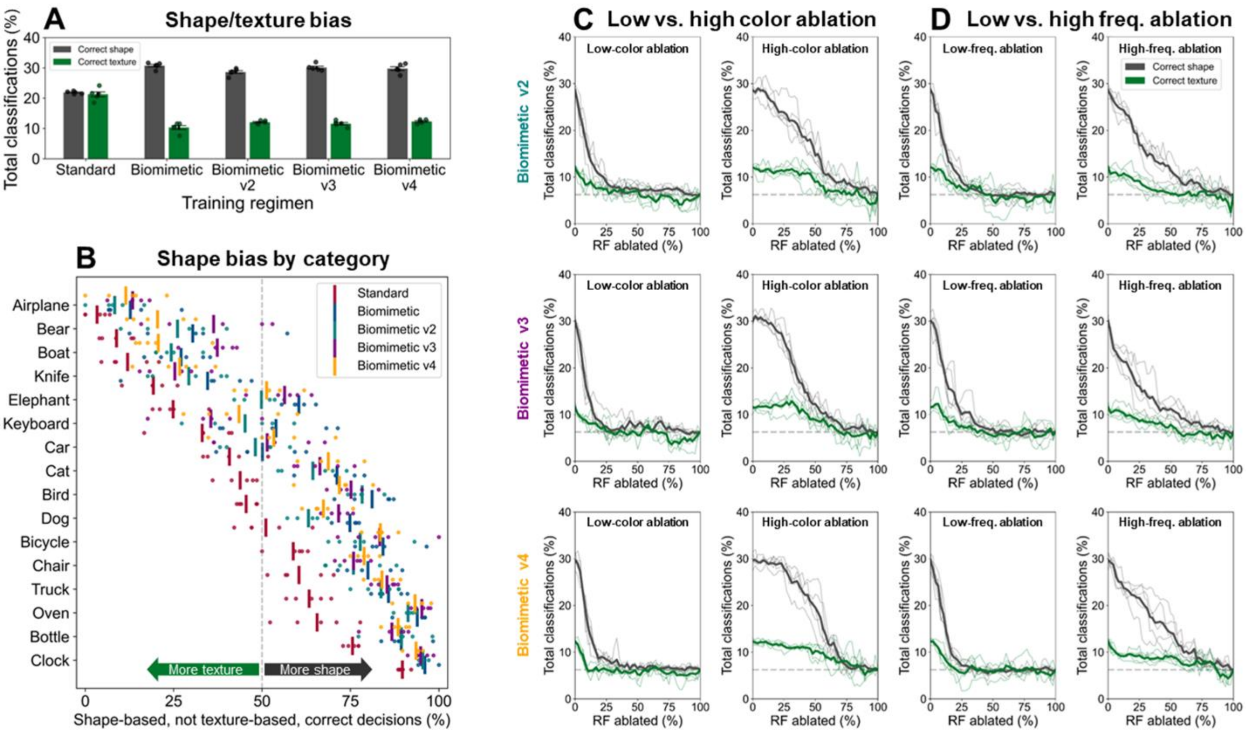

Supplementary Fig. 17. Reproduction and extension of Figure 2 in the main manuscript when utilizing biomimetic v2-v4 regimens within setting 1 (48 22x22 pixel RFs). **A.** Percentage of total classifications correct in terms of shape, correct in terms of texture, or incorrect. Error bars represent the standard error across the five different training runs, and dots depict results of each individual run. **B.** Percentage of shape-based correct classifications, as opposed to texture-based correct classifications, for each of the 16 different super-classes used. Shown here are results of the five individual training runs (dots), along with their means (bars). **C&D.** Shape/texture bias as a function of the proportion of ablated units with lowest color, highest color, lowest frequency, and highest frequency (from left to right) tuning. Depicted are the five training runs (thin lines) as well as their means (thick lines).

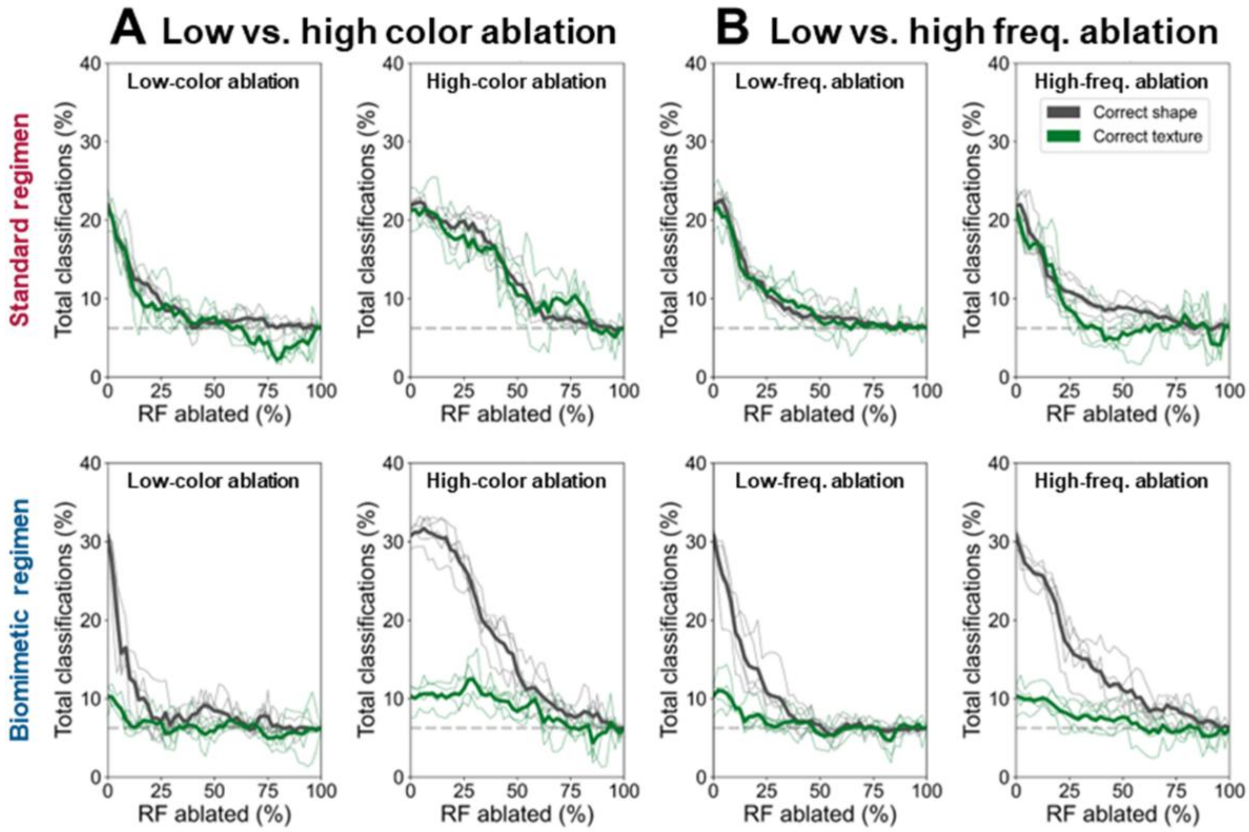

Supplementary Fig. 18. Results of unit ablation on shape/texture bias of setting 1 when carried out both with regard to the color and frequency metrics. The shape/texture bias is depicted as a function of the proportion of ablated units with lowest color, highest color, lowest frequency, and highest frequency (from left to right) tuning. Depicted are the five training runs (thin lines) as well as their means (thick lines).

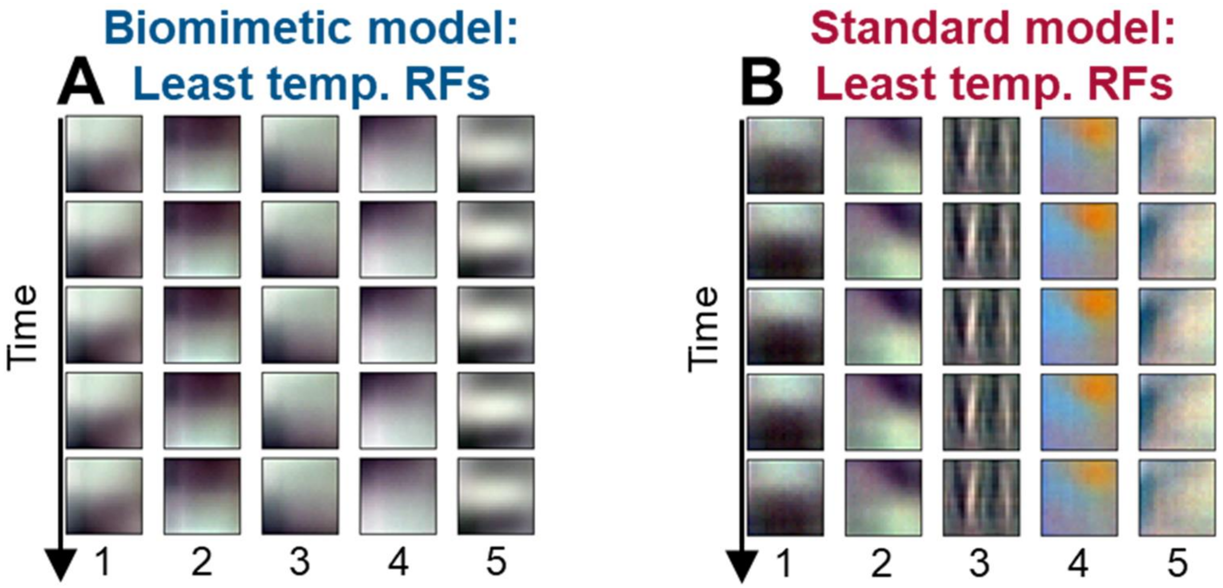

Supplementary Fig. 19. Visualization of the five least temporally varied receptive fields of the standard and biomimetic models (based on the first out of five training runs).

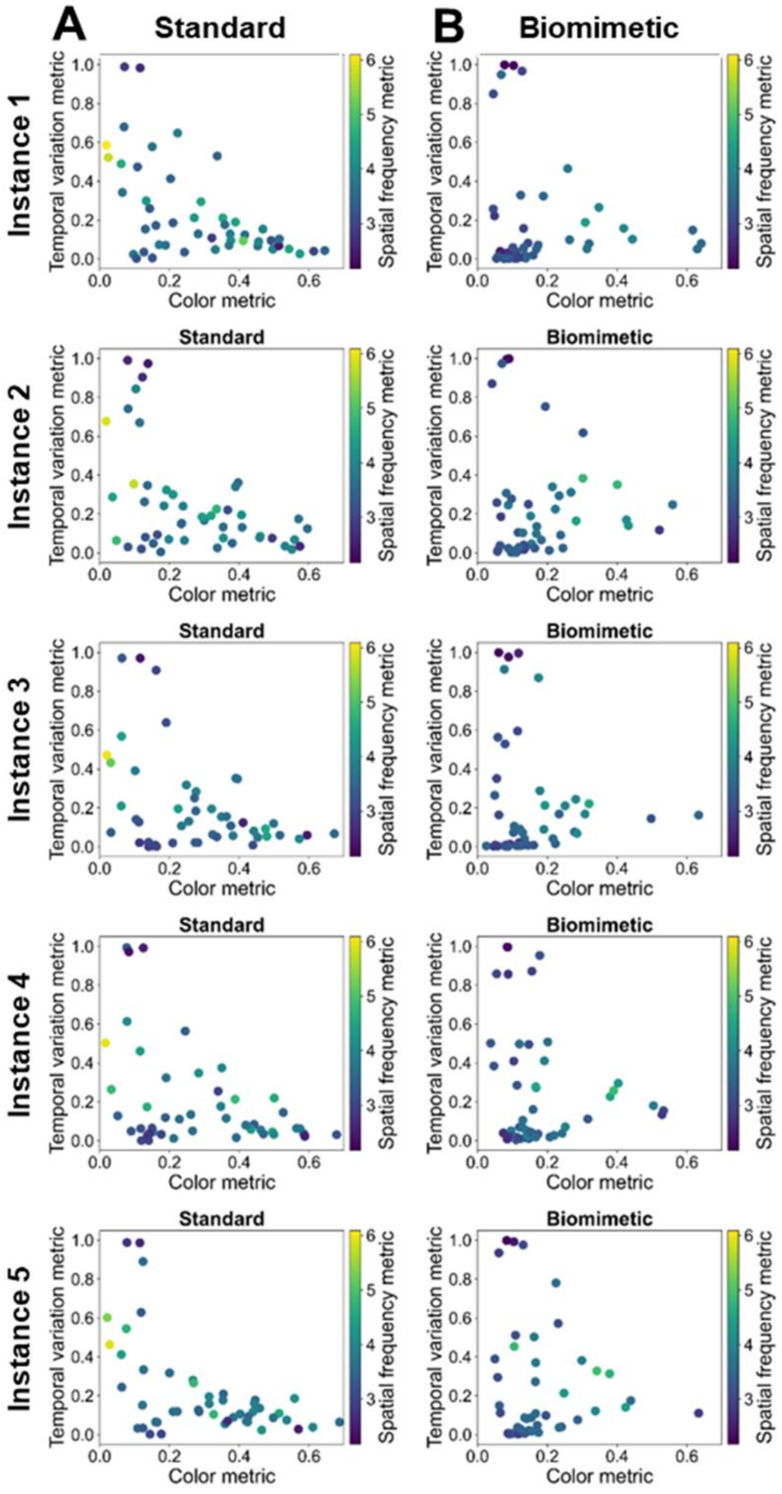

Supplementary Fig. 20. Depiction of joint RF metric distributions of our 3D CNNs across all five training runs with different random initializations (the first run shown in Figures 4C & 4F). Depicted is the relationship between temporal RF properties (using the temporal variation metric) and spatial RF characteristics (using the color and spatial frequency metrics used before) of both the biomimetic and standard model.

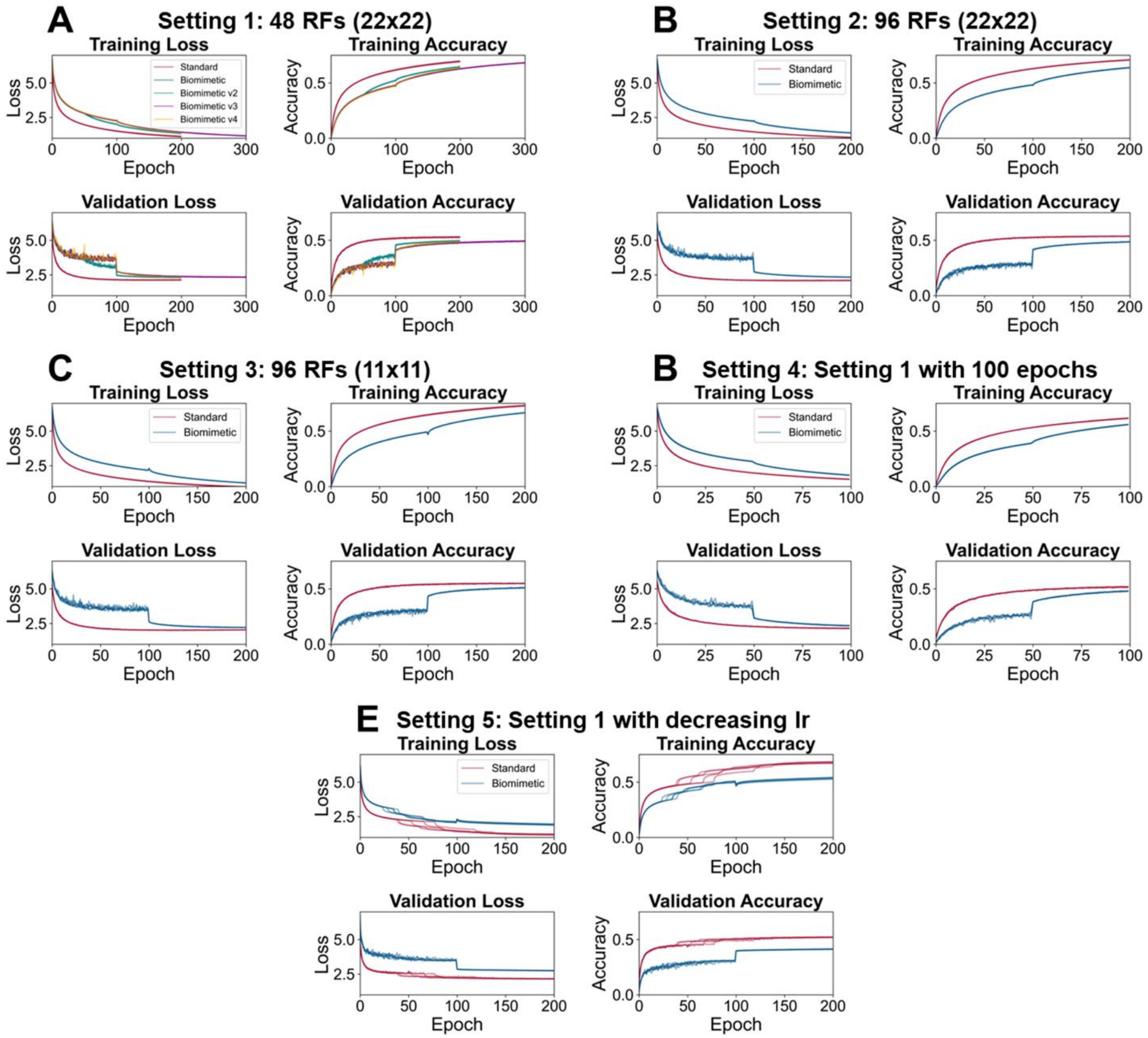

Supplementary Fig. 21. Depiction of training and validation loss / accuracy across the different settings and regimens used for training our 2D networks. Depicted are superimposed results of the five individual network training runs.

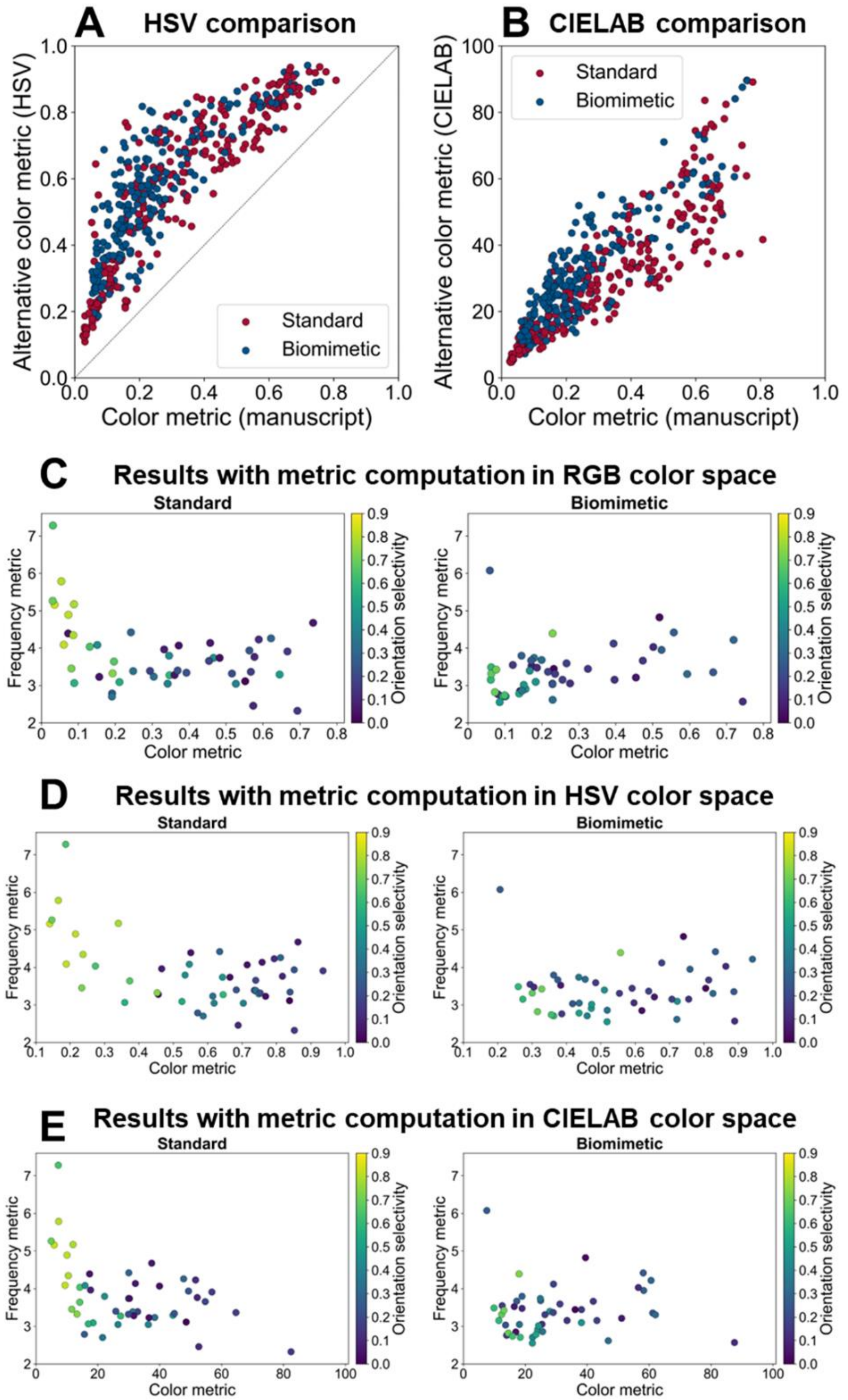

Supplementary Fig. 22. Further examination of our color metric. **A&B.** Relationship between individual RF characteristics based on our color metric (x-axis) and alternative color metrics based on the HSV and CIELAB color spaces (y-axes). Depicted are data corresponding to individual receptive fields across the 5 individual training runs for our main setting (setting 1). **C-E.** Scatter plots depicting the joint frequency and color coding of individual RFs, when using the original RGB-based, the HSV-based, or the CIELAB-based metric.

Supplementary Table 1. Architectural details of the 3D-CNN used in this study.

Input shape	32 (temporal) x 112 (spatial) x 112 (spatial) x 3 (color)
**Conv3D #1**	Number of kernels: 48<br>Kernel size: 5 (temporal) x 22 (spatial) x 22 (spatial)<br>Strides: 1 (temporal) x 4 (spatial) x 4 (spatial)<br>Activation function: ReLU
Batch Normalization	
Max Pooling 3D	Pool Size: 3 (temporal) x 3 (spatial) x 3 (spatial)<br>Strides: 2 (temporal) x 2 (spatial) x 2 (spatial)
**Conv3D #2**	Number of kernels: 256<br>Kernel size: 5 (temporal) x 5 (spatial) x 5 (spatial)<br>Strides: 1<br>Activation function: ReLU
Batch Normalization	
Max Pooling 3D	Pool Size: 3 (temporal) x 3 (spatial) x 3 (spatial)<br>Strides: 2
**Conv3D #3**	Number of kernels: 384<br>Kernel size: 3 (temporal) x 3 (spatial) x 3 (spatial)<br>Strides: 1<br>Activation function: ReLU
**Conv3D #4**	Number of kernels: 384<br>Kernel size: 3 (temporal) x 3 (spatial) x 3 (spatial)<br>Strides: 1<br>Activation function: ReLU
**Conv3D #5**	Number of kernels: 256<br>Kernel size: 3 (temporal) x 3 (spatial) x 3 (spatial)<br>Strides: 1<br>Activation function: ReLU
Batch Normalization	
Max Pooling 3D	Pool Size: 3 (temporal) x 3 (spatial) x 3 (spatial)<br>Strides: 2
Flatten	
**Fully-connected #1**	Kernels: 4096<br>Activation function: ReLU
Dropout	Dropout rate: 0.5
**Fully-connected #2**	Kernels: 4096<br>Activation function: ReLU
Dropout	Dropout rate: 0.5
**Fully-connected #3**	Kernels: 600 (= number of classes)<br>Activation function: Softmax
